# The relationship between visit-to-visit variability in blood pressure and incidence of metabolic syndrome: a general population-based cohort study in Korea

**DOI:** 10.1186/s40885-019-0117-9

**Published:** 2019-05-15

**Authors:** Hyung Tak Lee, June Namgung, Young-Hyo Lim, Hwan-Cheol Park, Jin-Kyu Park, Jinho Shin

**Affiliations:** 1 Departments of Internal Medicine, Gumdan Top General Hospital, Incheon, Republic of Korea; 2 Division of Cardiology, Department of Internal Medicine, Inje University Ilsan Paik Hospital, Goyang, Republic of Korea; 3 Division of Cardiology, Departments of Internal Medicine, Hanyang University, College of Medicine, 222 Wangsimni-ro Sungdong-gu, 133-792 Seoul, Republic of Korea

**Keywords:** Metabolic syndrome, Visit-to-visit variability, Blood pressure3

## Abstract

**Background:**

Previous studies demonstrated that visit-to-visit variability of blood pressure (BP) has significant relationship with cardiovascular disease. Visit-to-visit variability in BP might have prognostic value for cardiovascular disease. The aim of this study is to evaluate the effect of visit-to-visit variability in BP on development of metabolic syndrome in general population without cardiovascular disease, diabetes mellitus, metabolic syndrome, and BP medication.

**Method:**

We used data from the Korean Genome Epidemiology Study conducted by the Korean Centers for Disease Control and Prevention. All cohorts who were followed first 3 periods formed the basis of the study sample, which consisted of 7195 people. Of these samples, 3431 subjects who had cardiovascular disease, diabetes mellitus, or metabolic syndrome were excluded, and 312 subjects who were using antihypertensive medication in first 3 periods were excluded. Our final study sample consisted of 3452 cohorts.

**Results:**

The mean age was 53.5 (8.25) years. The proportion of male was 50.2%. Average follow-up duration was 5.91 (0.17) years. In generalized estimating equation, the development of metabolic syndrome was associated with mean systolic BP (SBP) (Odd ratio (OR) 1.042, 95% confidence interval (CI) 1.035–1.048, *p* < 0.001), mean diastolic BP (DBP) (OR 1.058, 95% CI 1.049–1.069, *p* < 0.001), standard deviation (SD) of SBP (OR 1.036, 95% CI 1.017–1.055, *p* < 0.001), SD of DBP (OR 1.053, 95% CI 1.027–1.080, *p* < 0.001), and coefficient of variation (CV) of DBP (OR 1.025, 95% CI 1.005–1.046, *p* = 0.016) after adjusted for age, sex, and metabolic syndrome component. When mean SBP, mean DBP, SBP variability, and DBP variability were entered all together in the analysis model, SD of DBP (OR 1.033, 95% CI 1.003–1.063, *p* = 0.030) and CV of DBP (OR 1.027, 95% CI 1.004–1.051, *p* = 0.020) were significantly associated with the development of metabolic syndrome.

**Conclusion:**

In general population without cardiovascular disease, diabetes mellitus, metabolic syndrome, and BP medication, SD of DBP and CV of DBP was associated with the development of metabolic syndrome. Visit-to-visit variability in DBP might be helpful for the prediction of future metabolic syndrome development.

## Background

The impact of increased blood pressure (BP) on cardiovascular disease is well established. In a clinical setting, the prognostic value of BP is generally estimated by measuring mean BP. On the other hand, previous studies demonstrated that visit-to-visit variability in BP also has significant relationship with cardiovascular disease [1–3]. In 1997, Suchy-Dicey et al. showed that higher visit-to-visit variability in BP is associated with increased risk for coronary artery disease during 11.6 years of follow-up [4]. In 2010, Rothwell et al., showed that increased visit-to-visit variability in BP is significant predictor of stroke [5, 6]. More recent studies showed that visit-to-visit variability in BP is associated with all-cause mortality [7]. However, exact mechanism by which visit-to-visit variability in BP is related with cardiovascular risk remains unclear.

Previous studies showed that metabolic syndrome is precursor of cardiovascular disease. A previous meta-analysis documented that metabolic syndrome is associated with increased risk for cardiovascular mortality, myocardial infarction, and stroke [8]. Another meta-analysis study, in which analyzed longitudinal studies, revealed that metabolic syndrome is significantly related to the cardiovascular event, coronary heart disease, and cardiovascular death [9]. Therefore, metabolic syndrome would be prodrome of cardiovascular disease. In this study, we hypothesized that the relationship between visit-to-visit variability in BP and cardiovascular disease is mediated by metabolic syndrome.

The aim of this study is to evaluate the effect of visit-to-visit variability in BP on development of metabolic syndrome in general population without cardiovascular disease, diabetes mellitus, metabolic syndrome, and BP medication.

## Methods

### Cohorts

**Fig. 1:**
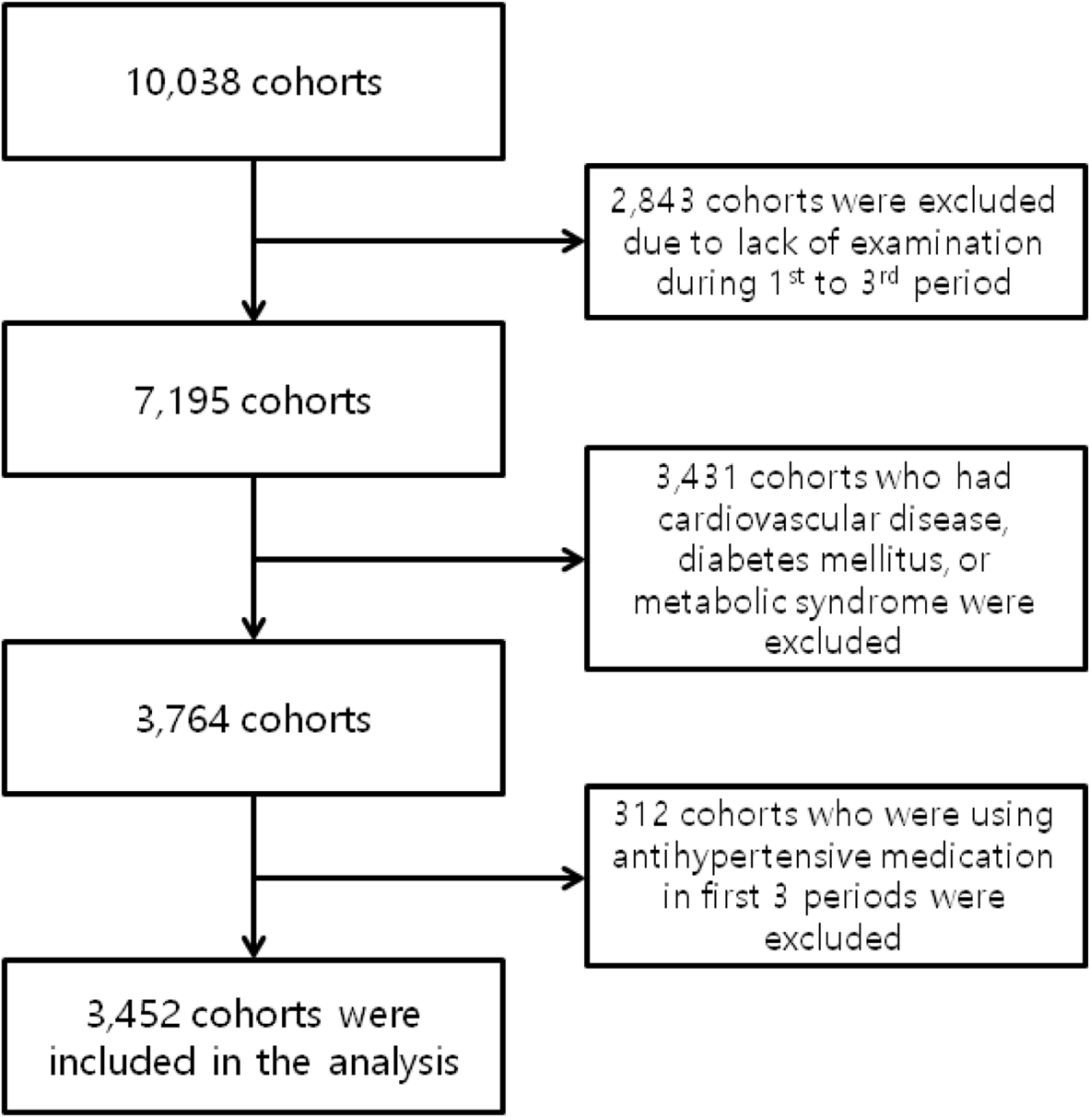
Flow chart of cohorts selection

We used data from the Korean Genome Epidemiology Study (KoGES) conducted by the Korean Centers for Disease Control and Prevention. KoGES started in 2001 in two cities, and is an ongoing prospective study involving a biennial examination. Ansung city and Ansan city represented rural and urban communities, respectively. Until now, KoGES has six periods. In first period, a total 10,038 cohorts were recruited. In Ansan, a total of 5020 subjects were recruited by telephone calls. The telephone calls were made to 10,957 randomly selected local telephone numbers requesting cohort participation. In Ansung, a total of 5018 subjects were recruited by using mailing, door-to-door visits, and telephone solicitations within five randomly selected local government regions (termed Myons in Korea) of the 11 divisions in the district. All cohorts who were followed first 3 periods without omission of examination formed the basis of the study sample, which consisted of 7195 people. Of these samples, 3431 subjects who had cardiovascular disease, diabetes mellitus, or metabolic syndrome were excluded, and 312 subjects who were using antihypertensive medication in first 3 periods were excluded. Our final study sample consisted of 3452 cohorts (Fig. 1).

### Follow-up

The cohort examinations were performed approximately 2 years interval. The first period, second period, third period, fourth period, fifth period, and sixth period were conducted from 2001 to 2003, 2003–2005, 2005–2006, 2007–2009, 2009–2010, and 2011–2012, respectively. We divided study time into two parts. From first period to third period, mean BP and visit-to-visit variability were taken, and from fourth to sixth period, subsequent follow-up was obtained. Average follow-up duration was 5.91 (0.17) years.

### Measurements

Health examination at third period was used as baseline examination. Participants were seated for at least 5 min before BP measurement. BP measurements were repeated after a 5 min interval. The arithmetic mean value of the two readings was used for analysis. Mercurial sphygmomanometers were used (CK-101, CHIN KOU Medical Instrument Co. Ltd., Taipei, Taiwan). The use of antihypertensive medication was assessed by an interviewer-administered questionnaire at baseline and at every visit in both the Ansan and Ansung cohorts. Blood samples of all participants were collected from the antecubital vein after at least 8 h of fasting. Enzymatic methods were applied to measure total cholesterol, high-density lipoprotein cholesterol, triglycerides, creatinine, and glucose (ADVIA 1650, Bayer Diagnostics, Tarrytown, NY, USA).

The metabolic syndrome was defined as subjects with three or more of the following criteria: 1) Waist circumference ≥ 90 cm in men and ≥ 80 cm in women; 2) Serum triglycerides levels of ≥150 mg/dL or undergoing drug treatment for elevated serum triglycerides levels; 3) High-density lipoprotein cholesterol levels of < 40 mg/dL in men and < 50 mg/dL in women or undergoing drug treatment for low High-density lipoprotein cholesterol; 4) BP of at least 130/85 mmHg or undergoing antihypertensive drug treatment due to a history of hypertension; or 5) fasting blood glucose level of ≥100 mg/dL or undergoing drug treatment for elevated fasting blood glucose level [10].

### Statistical analysis

All continuous variables were reported as mean values with standard deviations (SD) and categorical variables were presented as numbers and percentages. Visit-to-visit variability in BP was quantified using the standard deviation (SD) and coefficient of variation (CV). Logistic regression with generalized estimating equation models were used to test for the influence of BP variability on the prevalence of metabolic syndrome during follow-up. Baseline covariates used to obtain adjusted odds ratios in generalized estimating equation models were age, sex, region, body mass index, creatinine, waist circumference, total cholesterol, triglyceride, high-density lipoprotein cholesterol, fasting glucose and smoking. Statistical analysis was performed using PASW 18.0 (SPSS, Chicago, IL, USA). A *p* < 0.05 (two-tailed) was considered to indicate a statistically significant result.

## Results

The mean age of the subjects was 53.5 (5.25) years, and the proportion of males was 50.2%. Of the total 3452 subjects, 2056 subjects were urban residents. The mean systolic BP (SBP) was 111.5 (12.3) mmHg, and the mean diastolic BP (DBP) were 74.8 (8.18) mmHg. The SD of SBP (SD SBP) and SD of DBP (SD DBP) were 7.39 (4.462) mmHg and 5.38 (3.123) mmHg, respectively. The CV of SBP (CV SBP) and CV of DBP (CV DBP) were 0.065 (0.0369) and 0.072 (0.0409). Further descriptive data are displayed in Table 1.

**Table 1:** General characteristics of cohorts

Variable	Total	Male	Female	P
Number	3452	1734	1718	
Age, years	53.5 (5.25)	54.3 (8.58)	52.7 (7.81)	< 0.001
Urban, (%)	2056 (59.6)	1016 (58.6)	1040 (60.5)	0.245
Body mass index, kg/m^2^	23.2 (2.61)	23.1 (2.54)	23.4 (2.68)	< 0.001
Waist, cm	80.0 (7.24)	81.6 (6.51)	78.4 (7.59)	< 0.001
Hypertension, (%)	186 (5.4)	130 (7.5)	56 (3.3)	< 0.001
Glucose, mg/dL	88.2 (8.87)	90.22 (9.53)	86.1 (7.64)	< 0.001
Total cholesterol, mg/dL	188.9 (33.43)	186.0 (33.04)	191.81 (33.59)	< 0.001
HDL cholesterol, mg/dL	47.2 (10.63)	45.74 (10.71)	48.70 (10.35)	< 0.001
Triglyceride, mg/dL	108.9 (60.31)	120.1 (69.67)	97.6 (46.44)	< 0.001
HbA1c, %	5.3 (0.40)	5.3 (0.43)	5.3 (0.37)	0.657
Blood urea nitrogen, mg/dL	15.2 (4.08)	16.0 (4.18)	14.5 (3.83)	< 0.001
Creatinine, mg/dL	0.96 (0.142)	1.\ (0.119)	0.86 (0.093)	< 0.001
Smoking, packyear	10.0 (17.04)	19.7 (19.60)	0.2 (2.60)	< 0.001
Mean SBP, mmHg	111.5 (12.3)	113.7 (11.77)	109.34 (12.44)	< 0.001
Mean DBP, mmHg	74.8 (8.18)	76.9 (7.99)	72.6 (7.81)	< 0.001
SD SBP, mmHg	7.39 (4.462)	7.47 (4.531)	7.31 (4.393)	0.304
SD DBP, mmHg	5.38 (3.123)	5.44 (3.146)	5.31 (3.098)	0.230
CV SBP	0.065 (0.0369)	0.065 (0.0371)	0.066 (0.0368)	0.346
CV DBP	0.072 (0.0409)	0.070 (0.0402)	0.073 (0.0415)	0.089

Values are presented as mean (SD) or numbers (percentages)

*HDL* high density lipoprotein, *HbA1c* glycosylated hemoglobin, *SBP* systolic blood pressure, *DBP* diastolic blood pressure, *SD* standard deviation, *CV* coefficient of variation

Table 2 summarizes adjusted odds ratios for the development of metabolic syndrome from the multivariable generalized estimating equation models. The analysis models were adjusted baseline covariates as described above, but not adjusted for the other BP variables. When unadjusted for other BP variables, mean SBP, mean DBP, SD SBP, SD DBP, and CV DBP have significant positive correlation with the prevalence of metabolic syndrome.

**Table 2:** Multivariable adjusted odds ratios for the development of metabolic syndrome from generalized estimating equation models unadjusted for other blood pressure variables

		Odds ratio (95% CI)	*p*
Model 1	Mean SBP, per 1 mmHg increase	1.042 (1.035–1.048)	< 0.001
Model 2	Mean DBP, per 1 mmHg increase	1.059 (1.049–1.069)	< 0.001
Model 3	SD SBP, per 1 mmHg increase	1.036 (1.017–1.055)	< 0.001
Model 4	SD DBP, per 1 mmHg increase	1.053 (1.027–1.080)	< 0.001
Model 5	CV SBP, per 0.01 increase	1.022 (0.998–1.047)	0.075
Model 6	CV DBP, per 0.01 increase	1.025 (1.005–1.046)	0.016

Baseline covariates are age, sex, region, body mass index, waist circumference, total cholesterol, triglyceride, high-density lipoprotein cholesterol, fasting glucose, and smoking

Model 1 included mean SBP and baseline covariates

Model 2 included mean DBP and baseline covariates

Model 3 included SD SBP and baseline covariates

Model 4 included SD DBP and baseline covariates

Model 5 included CV SBP and baseline covariates

Model 6 included CV DBP and baseline covariates

*CI* confidence interval, *SBP* systolic blood pressure, *DBP* diastolic blood pressure, *SD* standard deviation, *CV* coefficient of variation

Table 3 shows the odds ratios for the development of metabolic syndrome from generalized estimating equation models adjusted for other blood pressure variables. After adjustment for mean BP and other BP variables, SD DBP and CV DBP have positive relationship with the prevalence of metabolic syndrome. However, the relationships of SD SBP and CV SBP with the prevalence of metabolic syndrome were not significant.

**Table 3:** Multivariable adjusted odds ratios for the development of metabolic syndrome from generalized estimating equation models adjusted for other blood pressure variables

	Odds ratio (95% CI)	*p*
Model 1		
Mean SBP, per 1 mmHg increase	1.041 (1.034–1.048)	< 0.001
SD SBP, per 1 mmHg increase	1.006 (0.987–1.025)	0.544
Model 2		
Mean DBP, per 1 mmHg increase	1.057 (1.046–1.067)	< 0.001
SD DBP, per 1 mmHg increase	1.035 (1.009–1.061)	0.009
Model 3		
Mean SBP, per 1 mmHg increase	1.041 (1.035–1.048)	< 0.001
CV SBP, per 0.01 increase	1.008 (0.985–1.032)	0.493
Model 4		
Mean DBP, per 1 mmHg increase	1.059 (1.049–1.070)	< 0.001
CV DBP, per 0.01 increase	1.029 (1.008–1.049)	0.006
Model 5		
Mean SBP, per 1 mmHg increase	1.033 (1.020–1.045)	< 0.001
Mean DBP, per 1 mmHg increase	1.014 (0.996–1.033)	0.126
SD SBP, per 1 mmHg increase	0.994 (0.973–1.015)	0.562
SD DBP, per 1 mmHg increase	1.033 (1.003–1.063)	0.030
Model 6		
Mean SBP, per 1 mmHg increase	1.032 (1.020–1.045)	< 0.001
Mean DBP, per 1 mmHg increase	1.017 (0.999–1.036)	0.069
CV SBP, per 0.01 increase	0.993 (0.967–1.019)	0.570
CV DBP, per 0.01 increase	1.027 (1.004–1.051)	0.020

Baseline covariates are age, sex, region, body mass index, waist circumference, total cholesterol, triglyceride, high-density lipoprotein cholesterol, fasting glucose, and smoking

Model 1 included mean SBP, SD SBP, and baseline covariates

Model 2 included mean DBP, SD DBP, and baseline covariates

Model 3 included mean SBP, CV SBP, and baseline covariates

Model 4 included mean DBP, CV DBP, and baseline covariates

Model 5 included mean SBP, mean DBP, SD SBP, SD DBP, and baseline covariates

Model 6 included mean SBP, mean DBP, CV SBP, CV DBP, and baseline covariates

*CI* confidence interval, *SBP* systolic blood pressure, *DBP* diastolic blood pressure, *SD* standard deviation, *CV* coefficient of variation

**Fig. 2:**
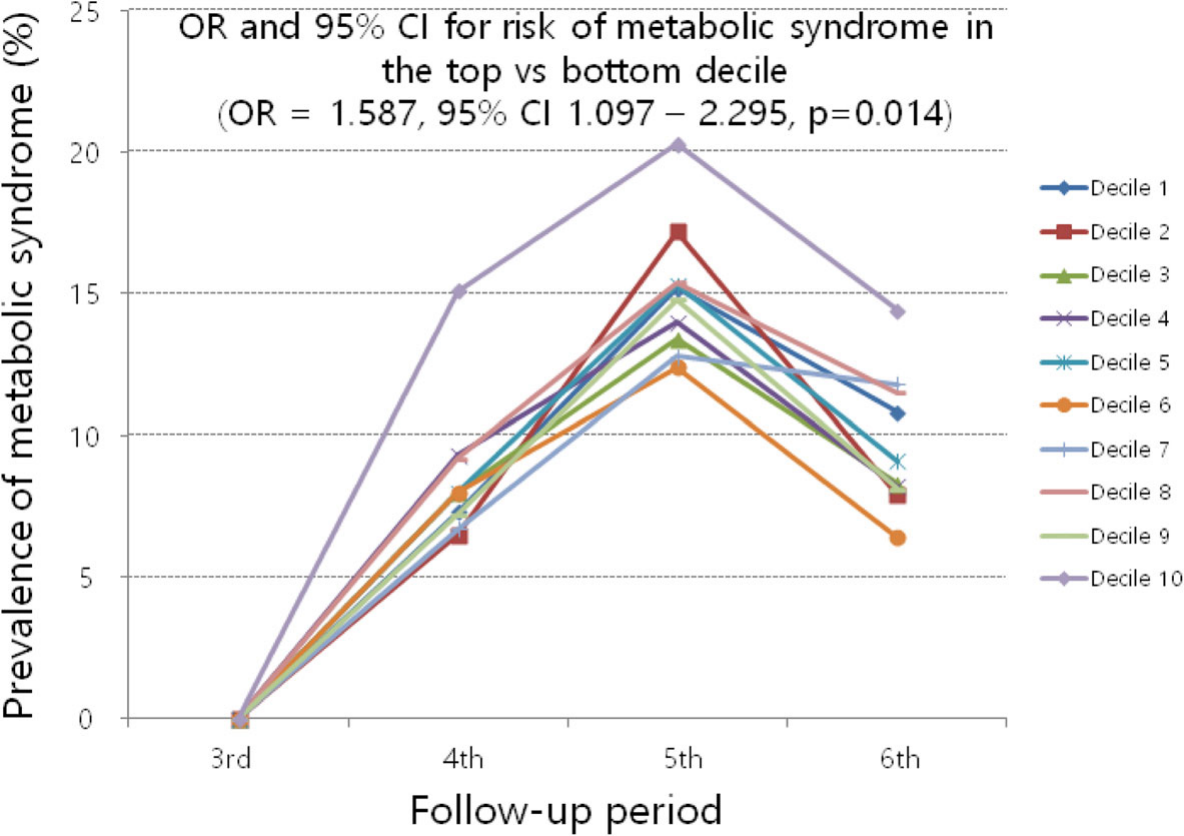
Prevalence of metabolic syndrome during follow-up by decile of SD DBP. Statistical values were derived from generalized estimating equation adjusted for age, sex, region, body mass index, waist circumference, total cholesterol, triglyceride, high-density lipoprotein cholesterol, fasting glucose, smoking, mean SBP, mean DBP, and SD SBP. OR, odds ratio; CI, confidence interval; SD, standard deviation; DBP, diastolic blood pressure; SBP, systolic blood pressure

**Fig. 3:**
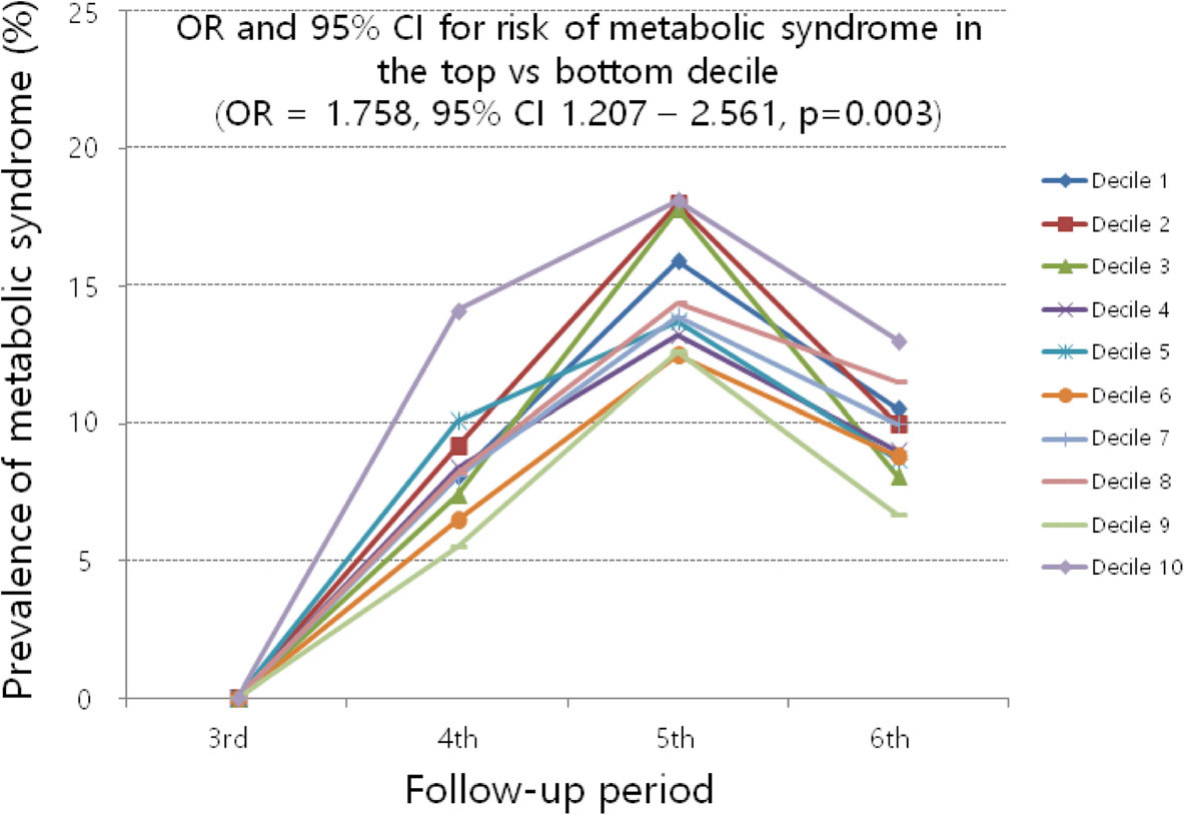
Prevalence of metabolic syndrome during follow-up by decile of CV DBP. Statistical values were derived from generalized estimating equation adjusted for age, sex, region, body mass index, waist circumference, total cholesterol, triglyceride, high-density lipoprotein cholesterol, fasting glucose, smoking, mean SBP, mean DBP, and CV SBP. OR, odds ratio; CI, confidence interval; CV, coefficient of variation; DBP, diastolic blood pressure; SBP, systolic blood pressure

Figure 2 and Fig. 3 show the change in prevalence of metabolic syndrome during follow-up by decile of SD DBP and CV DBP. The overall prevalence of metabolic syndrome during fourth period, fifth period, and sixth period were 8.0, 14.7, and 9.5%, respectively. The prevalence of metabolic syndrome decreased after fifth period. This result might be caused by cohort effect. SD DBP and CV DBP were split into deciles, and odds ratios of top deciles were calculated in relation to the bottom deciles. Top deciles of SD DBP and CV DBP have significantly higher odds ratios for the development of metabolic syndrome than bottom deciles.

## Discussion

This study shows that in healthy subjects, visit-to-visit variability in DBP is associated with a development of metabolic syndrome. The association was independent of mean BP value and other metabolic syndrome component.

Several studies demonstrated that in at risk population, visit-to-visit variability in SBP is significantly associated with future cardiovascular morbidity and mortality. Hsieh et al. study, in which 2161 type 2 diabetes mellitus subjects were followed for 5 years, showed that visit-to-visit variability in SBP, visit-to-visit variability in DBP, and visit-to-visit variability in mean BP are significantly associated with all-cause mortality independent of mean BP [11]. Similarly, Okada et al. found that visit-to-visit variability in SBP is positively correlated with diabetic nephropathy and atherosclerosis in patients with type 2 diabetes mellitus [12, 13]. In contrast, our study showed that visit-to-visit variability in DBP is significantly associated with future development of metabolic syndrome in healthy population independent of mean BP, whereas, visit-to-visit variability in SBP is not associated with development of metabolic syndrome. Such differences between our study and other previous studies might be caused by different population. Our study population is free from metabolic syndrome, diabetes mellitus, and cardiovascular disease. These results suggest that in healthy population, visit-to-visit variability in DBP might have more important role than visit-to-visit variability in SBP. However, the mechanisms for why only visit-to-visit variability in DBP should be related with the development of metabolic syndrome in healthy population remain unclear.

There are some potential explanations for the relationship between visit-to-visit variability in DBP and development of metabolic syndrome. Franklin et al. study, which is based on the Framingham Heart Study, suggested that obesity is more strongly related with increase in DBP than increase in SBP [14]. Similarly, Liu et al. showed that increasing body mass index is associated with increase in DBP [15]. These previous studies suggest that metabolic syndrome is related with DBP, and we can hypothesize that metabolic syndrome is related with visit-to-visit variability in DBP. In addition, our study population is relatively young. Old people have larger pulse pressure and lower diastolic pressure than young people [16]. In other word, young people have higher DBP than old people. A previous study suggested that high DBP is more prominent risk factor in younger people than older people [17]. Furthermore, high mean values have larger standard deviation than low mean values, and younger people may have higher mean DBP and larger visit-to-visit variability in DBP than older people. Therefore, in young population, visit-to-visit variability in DBP might have potent effect on the development of metabolic syndrome. In predominantly younger populations compared to previous studies, significant associations were present between visit-to-visit variability in DBP and metabolic syndrome.

This study excluded subjects who using BP medication. Recent studies suggested that the use of calcium-channel blockers lead to less visit-to-visit variability in BP than the use of angiotensin converting enzyme inhibitors, angiotensin 2 receptor antagonists, and beta-blocker [5, 6]. Therefore, our study shows the relationship between BP variability of unaffected by BP medication and development of metabolic syndrome. Because most of our study population is not hypertensive patient, we can hypothesize that the relationship between BP variability and development of metabolic syndrome is established in normal BP range. Previous studies suggested that increased arterial stiffness is associated with increased pulse pressure and increased BP variability [18, 19], and diabetes mellitus is related with increased arterial stiffness [20, 21]. In this viewpoint, increased arterial stiffness or increased BP variability might be not only product of metabolic syndrome but also cause of metabolic syndrome.

The present study should be interpreted in context of some limitations. The major limitation of this study is number of BP measurement. As the number of BP measurement increase, so do the reliability of visit-to-visit variability in BP [22, 23]. Moreover, the effect of visit-to-visit variability in BP on clinical outcome is increase as the number of BP measurement is increase [23]. In this study, visit-to-visit variability in BP was calculated from three measurement of BP. Although our sample size is relatively large, the effect of visit-to-visit variability in BP on clinical outcome might be underestimated. In addition, the present study only shows the association between visit-to-visit variability in BP and future development of metabolic syndrome. Underlying mechanism of the relationship between visit-to-visit variability in BP and the development of metabolic syndrome is not clear. Obesity and physical inactivity might be a part of the mechanism. Faramawi et al. suggested that obesity is related with increase in visit-to-visit variability in BP [24]. However, in this study, the relationships of visit-to-visit variability in DBP with change in BMI, waist circumference or other individual metabolic syndrome component were not significant (data not shown). BP is itself one component of metabolic syndrome. Therefore, the statistically insignificant relationship between visit-to-visit variability in DBP and change in obesity component might be caused by exclusion of BP component. Because the association is not necessarily causal, further study is warranted to determine the underlying mechanisms.

## Conclusion

In general population without cardiovascular disease, diabetes mellitus, metabolic syndrome, and BP medication, future development of metabolic syndrome is related with visit-to-visit variability in DBP, but not with visit-to-visit variability in SBP. These relationships are independent of mean BP and other metabolic syndrome component. Visit-to-visit variability in DBP might be predictive for future development of metabolic syndrome.

## Abbreviations

BP: Blood pressure
CV DBP: Coefficient of variation of diastolic blood pressure
CV SBP: Coefficient of variation of systolic blood pressure
CV: Coefficient of variation
DBP: Diastolic blood pressure
KoGES: Korean Genome Epidemiology Study
SBP: Systolic blood pressure
SD DBP: Standard deviations of diastolic blood pressure
SD SBP: Standard deviations of systolic blood pressure
SD: Standard deviations
